# Computational model of neuron-astrocyte interactions during focal seizure generation

**DOI:** 10.3389/fncom.2012.00081

**Published:** 2012-10-10

**Authors:** Davide Reato, Mario Cammarota, Lucas C. Parra, Giorgio Carmignoto

**Affiliations:** ^1^Department of Biomedical Engineering, The City College of the City University of New YorkNew York, NY, USA; ^2^Department of Experimental Biomedical Sciences, Institute of Neuroscience, Consiglio Nazionale delle Ricerche, University of PadovaPadova, Italy

**Keywords:** computational model, epilepsy, excitation/inhibition balance, neuron-astrocyte interaction, tripartite synapse

## Abstract

Empirical research in the last decade revealed that astrocytes can respond to neurotransmitters with Ca^2+^ elevations and generate feedback signals to neurons which modulate synaptic transmission and neuronal excitability. This discovery changed our basic understanding of brain function and provided new perspectives for how astrocytes can participate not only to information processing, but also to the genesis of brain disorders, such as epilepsy. Epilepsy is a neurological disorder characterized by recurrent seizures that can arise focally at restricted areas and propagate throughout the brain. Studies in brain slice models suggest that astrocytes contribute to epileptiform activity by increasing neuronal excitability through a Ca^2+^-dependent release of glutamate. The underlying mechanism remains, however, unclear. In this study, we implemented a parsimonious network model of neurons and astrocytes. The model consists of excitatory and inhibitory neurons described by Izhikevich's neuron dynamics. The experimentally observed Ca^2+^ change in astrocytes in response to neuronal activity was modeled with linear equations. We considered that glutamate is released from astrocytes above certain intracellular Ca^2+^ concentrations thus providing a non-linear positive feedback signal to neurons. Propagating seizure-like ictal discharges (IDs) were reliably evoked in our computational model by repeatedly exciting a small area of the network, which replicates experimental results in a slice model of focal ID in entorhinal cortex. We found that the threshold of focal ID generation was lowered when an excitatory feedback-loop between astrocytes and neurons was included. Simulations show that astrocytes can contribute to ID generation by directly affecting the excitatory/inhibitory balance of the neuronal network. Our model can be used to obtain mechanistic insights into the distinct contributions of the different signaling pathways to the generation and propagation of focal IDs.

## Introduction

The intracellular Ca^2+^ elevations occurring in cultured astrocytes in response to a glutamate challenge (Cornell-Bell et al., 1990) was the initial observation that hinted at the existence of a form of excitability in astrocytes based on cytosolic Ca^2+^ concentration changes. A few years later, Ca^2+^ elevations in astrocytes from both cell cultures (Parpura et al., 1994) and brain slices (Pasti et al., 1997) were observed to result in Ca^2+^ increases in nearby neurons mediated by astrocytic glutamate. Considering that astrocytes occupy non-overlapping spatial territories (Bushong et al., 2002; Halassa et al., 2007b) and that the processes of a single astrocyte can contact hundreds of synapses (Ventura and Harris, 1999), it was suggested that astrocyte-to-neuron communication may play a fundamental functional role in the brain. It was also found that astrocytes establish extensive contacts with cerebral blood vessels (Simard et al., 2003), which added further complexity to the functional role of neuron-to-astrocyte signaling. This neuron-astrocyte-blood-vessel signaling pathway was revealed to be central in neurovascular coupling, the process by which episodes of intense neuronal activity at restricted brain regions trigger local increases in cerebral blood flow to satisfy the energy demand of active neurons (Zonta et al., 2003; Mulligan and MacVicar, 2004; Gordon et al., 2008).

These pioneering results lead to the idea that astrocytes and neurons establish a bidirectional communication in the brain which may play fundamental roles in the modulation of synaptic transmission and plasticity (Carmignoto, 2000; Haydon, 2001).

Over the last decade numerous studies provided evidence for the ability of astrocytes to listen and talk to the synapse by exerting both excitatory and inhibitory actions on neurons (Araque et al., 1999; Brockhaus and Deitmer, 2002; Zhang et al., 2003; Pascual et al., 2005; Panatier et al., 2006; Serrano et al., 2006; Jourdain et al., 2007; Perea and Araque, 2007). These studies revolutionized our view of how the brain works. The processing of sensory information in the brain, which has been for many years considered to be based exclusively on neuronal communication, is now viewed as a product of the dynamic signals that neurons and astrocytes constantly exchange in the brain network. Such a bidirectional communication between neurons and astrocytes was conceptualized in the tripartite synapses in which the astrocyte composes with the pre-synaptic terminal and the post-synaptic target neuron, a third functional element of the synapse (Araque et al., 1999; Carmignoto, 2000; Halassa et al., 2007a; Perea et al., 2009).

The discovery that astrocytes are crucially involved in normal brain function raised the intriguing possibility that these cells may be involved also in brain dysfunction. The observation that glutamate released by astrocytes evokes episodes of synchronous activity in small groups of nearby neurons (Fellin et al., 2004, 2006), was the first clue suggesting that gliotransmission represent a relevant non-neuronal mechanism for neuronal synchrony, which may ultimately favor the generation of focal epileptiform activity (Kang et al., 2005; Tian et al., 2005). A new experimental protocol was recently developed by our group in rat enthorinal cortex (EC) slices in order to reproduce the spatial and temporal features of focal epileptiform discharges (Gomez-Gonzalo et al., 2010; Losi et al., 2010). In this model, a pharmacological stimulation of neurons from a restricted cortical region induces a propagating seizure-like ictal discharge (ID). The ability to emulate an epileptogenic focus allows us to study the early cellular events that take place during the generation of epileptiform activity as it arises at a focal site and propagates to the surrounding brain tissue. By using this experimental protocol we recently provided evidence that neurons engage astrocytes into an excitatory loop that pushes the neuronal network toward the ID generation threshold (Gomez-Gonzalo et al., 2010).

There are currently many computational models of seizures generation, development and cessation (Pitkänen et al., 2006). The level of description ranges from mean field models (Wendling et al., 2002; Suffczynski et al., 2004) to biophysically detailed models (Destexhe, 1998; Bazhenov et al., 2004; Traub et al., 2005). We used here a simplified approach in the description of the dynamics of single neurons and astrocytes. With this simplified dynamics we implemented a computational network model that allowed us to investigate the network mechanisms of focal ID generation and the role of astrocytes at the onset of the ID.

We found that the positive feedback provided by the astrocytes influences the dynamics of the system and favors the generation of epileptiform activities. The computational model quantitatively reproduces the spatial and temporal features of ID generation and propagation and provides mechanistic insights into the astrocyte contribution.

## Methods

### Neuron model

The computational model aims to reproduce the behavior of a brain network that in response to NMDA pulse stimulation generates a focal ID (Losi et al., 2010). The network consists of 320 excitatory and 80 inhibitory neurons randomly disposed and synaptically connected in a 2D configuration. As in our previous work (Reato et al., 2010), we used Izhikevich's model (Izhikevich, 2003) to describe the dynamics of single neurons. Briefly, the voltage dynamics of single neurons is characterized by four parameters: *a*, *b*, *c*, *d* as follows:

(1) $$\begin{array}{c} \frac{dv}{dt}=0.04v^{2}+5v+140-u+I=f(v,u)+I\\ \frac{du}{dt}=a\left(bv-u\right) \end{array}$$

With a reset of the dynamic variables *u, v* when a spike is generated:

(2) $$\text{if }v\geq 50\text{ mV},\text{ then }\{\begin{array}{l} v\leftarrow c\\ u\leftarrow u+d \end{array}$$

The choice of values for the four parameters defines different spiking behaviors. The parameters were chosen to reproduce the behavior of a regular spiking neuron for excitatory neurons (*a* = 0.02, *b* = 0.2, *c* = −65, *d* = 10) and of a fast spiking neuron for inhibitory neurons (*a* = 0.2, *b* = 0.26, *c* = −65, *d* = 0.5). The variable *I* represents the sum of the synaptic current and the external stimulation.

The synaptic currents mimic AMPA, NMDA, GABA_A_, and GABA_B_ receptor activation following (Izhikevich and Edelman, 2008). Briefly, the synaptic conductances are described by first-order linear kinetics, $\frac{dg_{x}}{dt}=-\frac{g_{x}}{\tau_{x}}+\sum_{j}s_{j}\delta \left(t-t_{f}\right)$ (where *x* = AMPA, NMDA, GABA_A_, GABA_B_) with, τ_AMPA_ = 1 ms, τ_NMDA_ = 2000 ms, τ_GABA_A__ = 6 ms, τ_GABA_B__ = 150 ms. Every time a pre-synaptic neuron *m* fires an action potential the conductance of the post-synaptic neurons increases instantaneously by *s* = *s*_exc_ and *s* = *s*_inh_ for pre-synaptic excitatory or inhibitory neurons respectively (*s*_exc_ = 0.001, *s*_inh_ = 0.01). The ratio of NMDA to AMPA receptors was set to be uniform at a value of 2, while GABA_B_ to GABA_A_ equal to 0.3 (*s*_exc_ = 0.002 for NMDA and *s*_inh_ = 0.003 for GABA_B_). The synaptic current of a post-synaptic neuron is then given by:

(3) $$\begin{array}{c} I_{\text{syn}}=I_{\text{exc}}+I_{\text{inh}}\\ I_{\text{exc}}=g_{\text{AMPA}}\left(v_{\text{exc}}-v\right)+g_{\text{NMDA}}\frac{{\left[\left(v+80\right)/60\right]}^{2}}{1+{\left[\left(v+80\right)/60\right]}^{2}}\\ I_{\text{inh}}=g_{{\text{GABA}}_{A}}\left(v_{\text{inh}}-v\right)+g_{{\text{GABA}}_{B}}\left(v_{\text{inh}}-v\right) \end{array}(v_{\text{exc}}-v)$$

Where *v* (function of time, Equation 1) is the voltage of the post-synaptic neuron and *v*_exc_ and *v*_inh_ are the reversal potentials for excitatory and inhibitory synapses. Here we chose *v*_inh_ = −90 mV, *v*_exc_ = 0 mV. Each neuron receives excitatory inputs from a square of maximum 48 neighbors, while inhibition from maximum eight neurons. Using these parameters a single excitatory pre-synaptic spike induces a depolarization of maximum ~0.1 mV, while an inhibitory pre-synaptic spike leads to maximum ~0.5 mV hyperpolarization. All the main parameters of the simulations (the *a*, *b*, *c*, *d* parameters describing the dynamics of single neurons for both excitatory and inhibitory neurons and the *s* parameters for synaptic connections) were selected from a normal distribution with standard deviation equal to 1% of the average value. To mimic the onset of an ID, a few parameters of the network were chosen in order to place the network in a hyperexcitable state. The excitability of excitatory neurons was slightly increased by injecting depolarizing currents (amplitude equal to 2), that could mimic the effects of 4-AP (a K^+^ channel blocker) used in the slice preparation. The high values chosen for both the conductance and the time constant of NMDA currents aim to reproduce the low Mg^2+^ experimental conditions. Without stimulation, both excitatory and inhibitory neurons are completely silent.

The NMDA stimulation that in experimental slice preparations evoked an ID was simulated in the model by depolarizing a set of neurons within a 7 × 7 square area above threshold for 500 ms (49 neurons). We refer to this as a simulated pulse (SimP). Alternatively, the NMDA pulses could have been simulated by activating NMDA currents. However, since we are interested in analyzing the effects on NMDA currents during the ID onset, this would have resulted in “stimulation artifacts” (the NMDA current induced by the pulse). Since we were also interested in studying the mechanisms leading to ID generation, the intensity of the stimulation was set to a value that not necessarily induced an ID in all the simulations (see Figure 3D).

In each simulation, nine SimPs were applied. In unsuccessful simulations, the average firing rate in the network increases during each SimP, but it rapidly comes back to zero between successive SimPs. An ID was considered to be successfully generated when the firing rate in neurons remains sustained above 1 Hz. The ID onset was then defined as the number of SimPs which starts this process.

The cessation of the ID was obtained by a modulation of the parameter *b* in a firing specific way. More specifically, we assumed that an elevated spiking activity decreases the excitability of single neurons. Possible physiological correlates of this event are the inactivation of Na^+^ channels (Bazhenov et al., 2004), the activation of Ca^2+^- or Na^+^-dependent K^+^ channels (Alger and Nicoll, 1980; Schwindt et al., 1989; Bazhenov et al., 2004; Timofeev et al., 2004) or the exhaustion of metabolic support (Yamada et al., 2001; Kirchner et al., 2006).

The equation used is:

(4) $$\frac{db}{dt}=-mR(t)+\left(b_{s}-b\right)$$

Where *R(t)* is the spike train of a single neuron low-pass filtered (time constant equal to 150 s), *m* is the coupling constant between the spiking activity and *b* (chosen here to be 15), and *b*_*s*_ the value of *b* in resting conditions (no spiking activity). The second term in the equation can be thought as a driving force to recover the normal neuronal functionality of the neuron, for example the metabolic support.

Because of the hyperexcitability of the network, i.e., neurons are firing intensively at ID onset, we had to integrate Izhikevich's equations with the method proposed in Izhikevich (2010) assuming the time step to be 1 ms:

(5) $$v(t+1)=\frac{v(t)+f(v(t),u(t))+g(t)E(t)+I}{1+g(t)}$$

Where *E*(*t*) = ∑(*g*_*i*_(*t*)*E*_*i*_)/*g*(*t*) with *g*(*t*) = ∑*g*_*i*_(*t*) (the total sum of conductances) and *E*_*i*_ = *v*_exc_, *v*_inh_ for excitatory and inhibitory connections, respectively. This method is efficient and stable even for large synaptic currents.

“Excitation” refers to the sum of excitatory currents (AMPA and NMDA) averaged across neurons, and similarly “inhibition” refers to the average summed inhibitory currents (GABA_A_ and GABA_B_). Excitatory and inhibitory firing rate indicate the firing rate of excitatory and inhibitory neurons, respectively. Where otherwise indicated, excitation, inhibition and firing rates of single simulations were always filtered with a moving average filter using a 50 ms time window for better visualization. Postictal refractory period was estimated as the time between the end of the seizure (average firing rate back to zero) and the time at which the *b* variable recovers to the 95% of the initial value.

Under the conditions described above, our computational model is able to generate a neuronal network activity which resembles several characteristics of experimental focal IDs (see later in the “Results”):

- the simulated ID originates from a small number of neurons in the network and propagates outside the focal area with a delay (Traub and Wong, 1982; Avoli et al., 2002);
- the simulated ID arises from an unbalance between inhibitory and excitatory activity at the focal area (Bradford, 1995; Ben-Ari, 2002);
- the simulated IDs have a cessation and a similar average duration (Jefferys, 1990; Traub et al., 1993; Pinto et al., 2005);
- the network enters into a period of postictal refractoriness (Jefferys, 1990);
- the peak in the firing rate of the excitatory and inhibitory neurons during simulated IDs is compatible with that measured in the *in vitro* experimental models.

Our model failed to reproduce the bursting behavior which characterizes the firing discharges in individual neurons and the two main phases in ID development, i.e., the initial tonic and the delayed clonic activity. However, the main focus in this computational model was to include astrocytes in the neuronal network and gain insights into how these non-neuronal cells can affect the equilibrium between excitation and inhibition in the network.

### Astrocyte model

We introduce here a simple representation of astrocytes interacting with a neuronal network. The parameters related to the ability of astrocytes to respond to neuronal activity with cytosolic Ca^2+^ elevations were captured from results obtained in experiments performed both in brain slices (Pasti et al., 1997; Porter and McCarthy, 1997) and in the living brain (Hirase et al., 2004; Wang et al., 2006; Kuga et al., 2011). To simulate the Ca^2+^ dynamics of a single astrocyte we used a framework similar to the Izhikevich neuron model. The equations represent a dynamical system of two variables [(Ca^2+^) and φ], without non-linear action potential or reset. The set of equation describing the Ca^2+^ concentration has the following form:

(6) $$\begin{array}{c} \frac{d\left[{\text{Ca}}^{2+}\right]}{dt}=-\varphi +\sum_{j}\sigma_{j}\delta \left(t-t_{f}\right)\\ \frac{d\varphi }{dt}=\alpha \left(\beta \left[{\text{Ca}}^{2+}\right]-\varphi \right) \end{array}$$

where φ is a recovery variable and σ_*j*_ is assumed here to be the neuronal input when an action potential is generated by the neuron *j*, since astrocytes respond to neuronal releases of glutamate (Pasti et al., 1997; Porter and McCarthy, 1997). Although the equations are dimensionless, the values σ_*j*_ where chosen to reproduce the pattern and amplitude of the Ca^2+^ elevations that are experimentally observed in astrocytes in response to neuronal activity (Porter and McCarthy, 1996; Pasti et al., 1997). The values of σ_*j*_ was chosen as been normally distributed with mean 0.00083 and standard deviation equal to 1% of the mean. Ca^2+^ concentration was restricted to be non-negative. Similarly to the dynamics described by the Izhikevich's single neuron, different values of α and β determine different behaviors (time constant of changes and coupling with the recovery variable). Here we chose α = 0.001 and β = 0.01. This choice was made to reproduce the slow time course of Ca^2+^ changes in astrocytes (Kawabata et al., 1996). When astrocytes were included in the whole network, these values were chosen to be normally distributed with a standard deviation equal to 1% of the mean.

To describe the release of astrocytic glutamate triggered by Ca^2+^ elevations, we considered a first order dynamics (low pass filters), with a release of glutamate that can occur only when Ca^2+^ reach a threshold (Pasti et al., 1997; Parpura and Haydon, 2000; Pasti et al., 2001):

(7) $$\begin{array}{c} \mu \frac{d\left[\text{glu}\right]}{dt}=\left\{ \begin{array}{l} -\left[\text{glu}\right]+\left(\left[{\text{Ca}}^{2+}\right]-{\left[{\text{Ca}}^{2+}\right]}_{\text{th}}\right)-\kappa \lambda \\ -\left[\text{glu}\right]-\kappa \lambda \end{array}\right.\begin{array}{c} \text{if}\left[{\text{Ca}}^{2+}\right]>{\left[{\text{Ca}}^{2+}\right]}_{\text{th}}\\ \text{otherwise} \end{array}\\ \eta \frac{d\lambda }{dt}=-\lambda +\left[\text{glu}\right] \end{array}$$

Where [Ca^2+^]_th_ = 0.0018 mM is the threshold for glutamate release, κ = 200 describes the coupling between the glutamate concentration [glu] and the recovery variable λ. Glutamate concentration was imposed to be non-negative. The time constants for the two variables were μ = 0.5 s and η = 10 s. The value of [Ca^2+^]_th_ was set based on available data showing that an increase in astrocytic Ca^2+^ of a few hundreds of nM was able to trigger glutamate release (Parpura and Haydon, 2000). Assuming a value of 200 nM for a single synapse (Nadkarni and Jung, 2003) and considering that astrocytes in our model receive inputs from a maximum of nine neurons, the threshold value can be determined by multiplying the value for the single synapse by the number of inputs, as considered in other studies (Wade et al., 2011).

**Figure 1:**
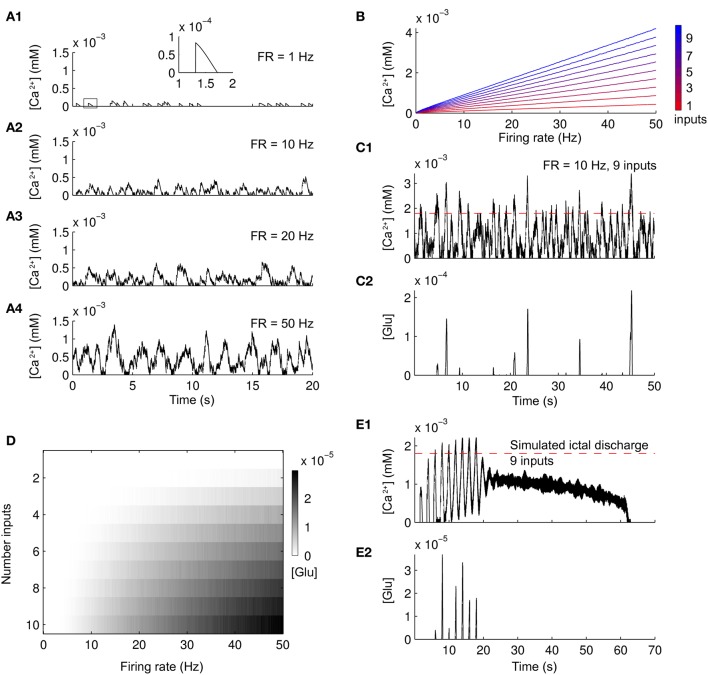
**Ca^2+^ changes and glutamate released from a simulated astrocyte in response to different patterns of neuronal firing. (A1–A4)** Ca^2+^ change in the simulated astrocyte induced by Poisson-distributed spike trains of increasing frequencies of an individual neuron. A single Ca^2+^ increase is outlined by the box in **(A1)** and expanded within the inset. **(B)** Summary of the Ca^2+^ concentration in the simulated astrocyte as a function of an increasing number of Poisson-distributed neuronal inputs (colorbar from red to blue) and increasing firing frequency. **(C1,C2)** Ca^2+^ change and glutamate released from the simulated astrocyte in response to a Poisson-distributed spike trains of nine inputs. Glutamate is released only when Ca^2+^ is above a threshold (red dashed line). **(D)** Summary of the glutamate released from the simulated astrocyte (gray scale colorbar) in response to the same neuronal stimulation as in **(B)**. **(E1,E2)** Ca^2+^ change and glutamate released from the simulated astrocyte during a simulated ID.

The set of parameters used for a single astrocyte reproduces basic features of Ca^2+^ dynamics and glutamate release in astrocytes. Increasing the input to an astrocyte, simulated as Poisson-distributed spike trains of increasing frequencies, leads to increasing Ca^2+^ concentrations (Figures 1A1–A4). The Ca^2+^ increase due to a single spike is less than 100 nM and lasts for about half a second (inset in Figure 1A1). These results are compatible with experimental evidences (Pasti et al., 1997; James et al., 2011) and previous computational models (Jefferys, 2003; Nadkarni and Jung, 2004, 2007; Wade et al., 2011). The linear dependence of Ca^2+^ increases as a function of simulated firing rate is reported in Figure 1B. Increasing the number of inputs by summing up Poisson-distributed spike trains (color scale from red to blue) also elevated Ca^2+^ concentrations. Since the release of glutamate due to the Ca^2+^ increases occurs only when Ca^2+^ is above a threshold, only strong activation can drive the release. As an example, nine spike trains at 10 Hz induced transient releases of glutamate (Figures 1C1,C2, Ca^2+^ threshold in red). Figure 1D summarizes the dependence of glutamate released by the astrocyte as a function of the firing rate and the number of inputs. For very low firing rates, there is no astrocytic glutamate release independently on the number of inputs. In the case of high firing rates, the release is linearly dependent on both the number of inputs and the firing rate. To further validate the parameters that we choose, we stimulated single astrocytes with a spike train from nine neurons from a simulated ID (see later in the “Results”). The neuronal activity leads to Ca^2+^ increases in the astrocyte (Figure 1E1) that caused a glutamate release (Figure 1E2) only after the second pulse (see also below). Interestingly, when the ID was fully developed, Ca^2+^ elevations reached a steady state value and glutamate was no longer released. It is known from experiments in cultures and in brain slices (Pasti et al., 1997; James et al., 2011) that upon intense stimulation the Ca^2+^ level in astrocytes increases rapidly and remains at an elevated steady-state value for tens of seconds (Figure 1E1). A single episode of glutamate release is experimentally observed only after the initial Ca^2+^ rise.

Astrocytes were included in the network with a 1:1 ratio with neurons. The ratio of glia to neurons increases in phylogenesis and is 1.65 in the human frontal cortex (Oberheim et al., 2006; Sherwood et al., 2006). Given that astrocytes account for about 50% of the total number of glial cells, a 1:1 ratio seems to represent an acceptable approximation. The input from neuronal activity, σ for each *m* astrocyte, was considered as the excitatory input from neurons firing *s*_exc_(*m*) (the excitatory component) in a 3 × 3 square (inputs from nine excitatory neurons). This choice was made considering that the feedback of astrocytes on neuronal activity is thought to be local with four to eight neuronal somata enveloped by a single astrocyte (Halassa et al., 2007b), but with the processes from a single astrocyte associated with up to 600 dendrites and many thousands of synapses (Bushong et al., 2002; Oberheim et al., 2006). The glutamate released by astrocytes was used as input to the same neurons to which the astrocyte is exposed. This glutamate generated NMDA currents in these neurons by activating the NMDA channel (in the same way than *s*_exc_). In some simulations (see “Results”) we considered the effects of inhibitory inputs from astrocytes. This was done by considering that the astrocytic response activate GABA_A_ receptors instead of NMDA (so simulating the effect of GABA release).

All the simulations were performed using MATLAB (Mathworks), and the code is available at www.neuralengr.com/code.

## Results

### Focal ID generation in entorhinal cortex slices

**Figure 2:**
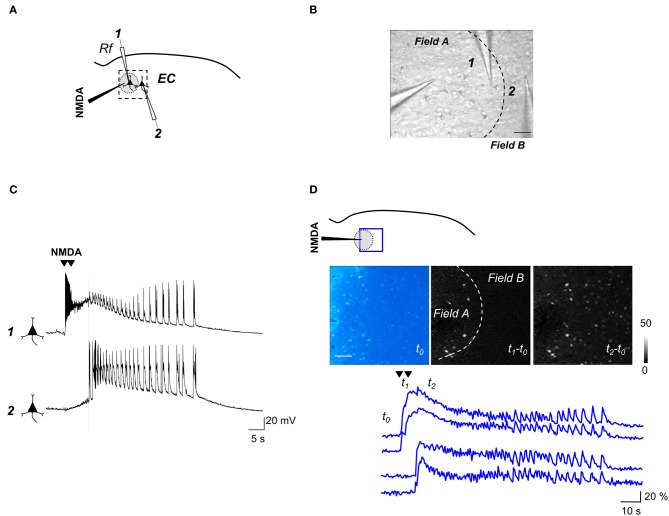
**Experimental protocol for inducing focal IDs in brain slices. (A)** Schematic of a dual voltage-clamp recording from EC deep layers pyramidal neurons (PyNs) with indication of the NMDA pipette and the patched neurons (1,2), one within the region directly activated by NMDA (gray circle) and the other immediately outside (~200 and ~350 μm from the NMDA pipette respectively). **(B)** DIC image of the area marked by the dashed box in **(A)**. Scale bar 100 μm. **(C)** Dual current-clamp recording from two PyNs during a focal ID evoked by a double NMDA pulse (arrowheads). The vertical dashed line marks the generation of ID in both cells. Note that in neuron 1 the direct NMDA response precedes and partially masks the ID onset. **(D)** Schematic of an experiment similar to that reported in **(A)** showing the region of Ca^2+^ signal imaging (blue box). The image of the basal OGB1 fluorescence in the EC (*t*_0_) and the difference images of the fluorescence signal change captured at different times (see lower traces) during an evoked ID (*t*_1_–*t*_0_; *t*_2_–*t*_0_) are reported. The NMDA-pipette is visible on the left. The dashed line in the image *t*_1_–*t*_0_ marks the region directly activated by NMDA. Scale bar 100 μm. Lower traces show the Ca^2+^ signal change from two neurons that were directly activated by NMDA (upper traces) and two other neurons, located outside the region directly activated by NMDA, that exhibited the ID only (lower traces). The timing that corresponds to the different images (*t*_0_, *t*_1_, and *t*_2_) is reported.

As we previously reported (Losi et al., 2010), an episode of neuronal hyperactivity can generate a focal ID in EC slices perfused with the K^+^ channel blocker 4-aminopyridine (4-AP) and low Mg^2+^. Figure 2 illustrates a typical ID that was generated in cortical layer V-VI by a double brief pressure pulse applied to an NMDA-containing glass pipette (Figures 2A,B). Dual patch-clamp recordings revealed that the firing in neurons located within the focus (Figure 2C, neuron 1) evolved into a focal ID with some delay after the NMDA double pulse. Following the ID generation at the focus (Figure 2A, gray circle), neurons outside the focus (<400 μm from the NMDA pipette tip) were also recruited and exhibited a similar pattern of action potential firing (Figure 2C, neuron 2). Given that the somatic Ca^2+^ change in neurons reflects faithfully the action potential firing in these cells, in slices loaded with the Ca^2+^ sensitive dye Oregon Green BAPTA1-AM (OG-B1-AM) we could monitor the activity of tens of neurons and follow how a focal ID is generated in the neuronal network. These experiments revealed that the NMDA stimulation evoked a rapid Ca^2+^ elevation in neurons located within the focal area, while neurons from the surrounding network were recruited into the ID only after a delay of 10.9 ± 0.8 s (30 IDs from 15 slices).

### Focal ID generation in the neuronal network model

**Figure 3:**
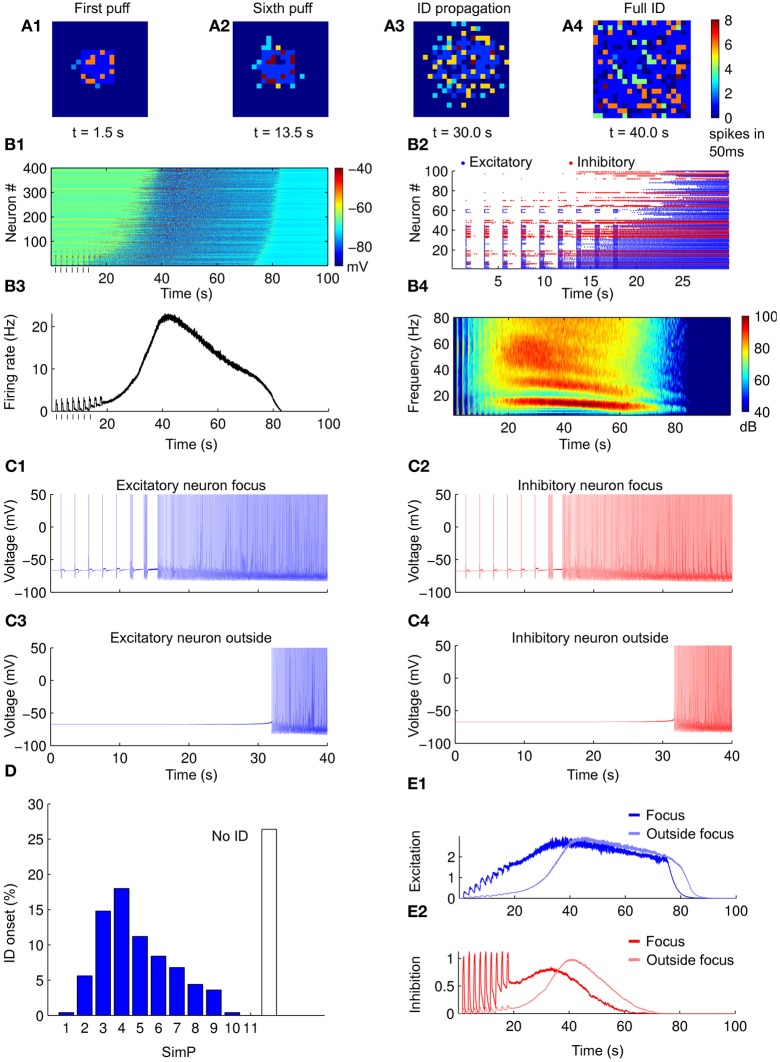
**Simulated IDs from a purely neuronal network. (A1–A4)** Pseudocolor plot of the number of action potentials calculated in 50 ms time windows at different times of the simulation. **(B1)** Membrane voltage (considered from −90 to −40 mV) for all the neurons in the network during a simulated ID. **(B2)** Raster plot of the spiking activity of a subpopulation of neurons in the network in the first 30 s of simulation (excitatory in blue and inhibitory in red). **(B3)** Average firing rate of the neurons in the network during a simulated ID. **(B4)** Spectrogam of the average firing rates for 40 simulations. Note the high frequency component corresponding to the firing of inhibitory neurons and the low one corresponding to excitatory neurons. **(C1–C4)** Examples of neuronal voltage traces of excitatory and inhibitory neurons within and outside the focus. **(D)** Histogram of ID threshold for 250 simulations. **(E1,E2)** Average value of excitation/inhibition (average post-synaptic excitatory currents) at the focus (blue/red), outside the focus (light blue/light red). Note that, differently from excitation, the inhibition does not accumulate after subsequent SimPs.

In the model we first examined how the neuronal network responds to a sequence of simulated NMDA pulses in the absence of astrocytes. To mimic the NMDA pulses at the focus, a depolarizing current pulse was injected for 500 ms in an area of 7 × 7 neurons (see “Methods”). The first SimP evoked robust spiking activity that remained restricted to neurons of the focus (Figure 3A1). Upon successive SimPs the firing activity spread from the focus to the surrounding neurons approximately 10 s after the SimP onset (Figures 3A1–A4,B1). The neuronal firing discharge remained high thereafter for tens of seconds (61 ± 2 s) before a sudden cessation (Figure 3B1). A postictal refractory period was observed with an average duration of 266 ± 1 s (see “Methods”). This pattern of activity resembles the focally evoked ID in slice preparations (see Figure 2D). A raster plot of the activity in a subpopulation of excitatory and inhibitory neurons within and outside the focus revealed that inhibitory neurons fire more intensively as compared to excitatory neurons during the SimPs, while the spiking activity in excitatory neurons increases with successive SimPs (Figure 3B2). The peak of the activity in the whole network was reached during the ID (Figure 3B3) and its spectrogram clearly revealed two main components corresponding to the different activity in excitatory and inhibitory neurons that fire at about 15 and 60 Hz, respectively (Figure 3B4). This pattern of activity in the two neuronal populations is consistent with experimental observations (Ziburkus et al., 2006). While both excitatory and inhibitory neurons at the focus were activated upon the initial stimulation (representative traces in Figures 3C1,C2), neurons outside the focus were recruited into the propagating ID with some delay (representative traces in Figures 3C3,C4).

### ID generation threshold

We consider as ID threshold the number of NMDA pulses that are needed to evoke an ID. This value is constant for a given slice (Gomez-Gonzalo et al., 2010), but it can vary for different slices. Simulations with different parameters (see “Methods”) showed that an ID could be generated in average by five SimPs and in more than 25% of cases no IDs could be evoked regardless the number of applied SimPs (Figure 3D, *n* = 250 simulations). The successive SimPs induced excitatory responses at the focus with increasing amplitude (Figure 3E1). The excitatory and inhibitory neurons outside the focus were not directly activated by the SimPs and increased their firing activity simultaneously, but with a marked delay (Figures 3E1,E2).

### Dynamics of excitation and inhibition at the focus explain ID generation

**Figure 4:**
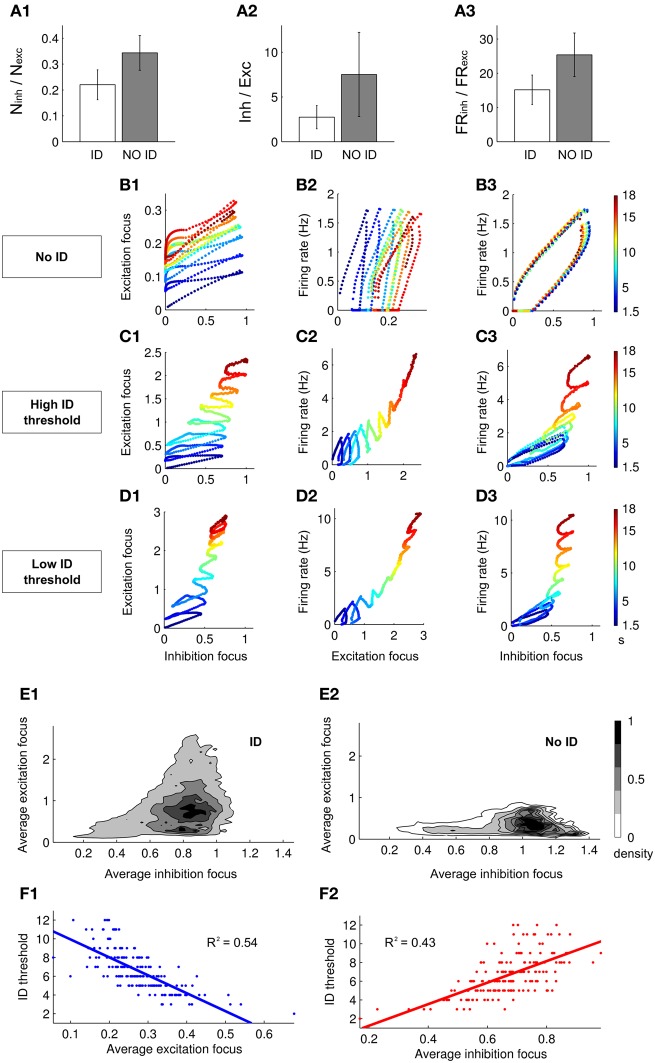
**Excitation–inhibition interplay during ID generation. (A1–A3)** Histograms showing the difference between the ratio of the number **(A1)**, the average synaptic currents **(A2)** and the firing rate **(A3)** between inhibitory and excitatory neurons when an ID is or is not generated (**A2** and **A3** are relative to the response to the first SimP). **(B1–B3,C1–C3,D1–D3)** Dynamic representation of the first 18 s of simulations in which an ID: **(B)** is not generated, **(C)** is evoked with a high threshold (five SimPs) and **(D)** is evoked with a low threshold (three SimPs). The dynamics of the network is represented as a point in the plane representing excitation and inhibition (in the focus, **B–D1**), the average firing rate of the network and excitation (at the focus **B–D2**) and the average firing rate of the network and inhibition (at the focus, **B–D2**). **(E1,E2)** Normalized density of dynamical points in the excitation/inhibition plane during the first seven SimPs (250 simulations) when an ID is generated **(E1)** or it is not generated **(E2)**. **(F1,F2)** Linear regression showing the correlation between the ID threshold and, respectively, the average excitation **(F1)** and inhibition **(F2)** in the focus.

We next investigated the interplay between excitation and inhibition in the genesis of the ID. We compared the simulations which successfully evoked an ID with those that failed to evoke an ID (in the different simulations excitatory and inhibitory neurons were randomly located within or outside the focus while maintaining their total number). For the cases in which an ID was successfully generated, we find that the ratios between the number of excitatory and inhibitory neurons, the average inhibitory and excitatory currents during the first SimP and the firing rate of inhibitory and excitatory neurons in the same time interval were lower compared to cases where an ID was not successfully generated (Figures 4A1–A3).

We next tested whether a different strength in either excitation or inhibition at the focus changed the efficacy of the SimP in generating an ID. We analyzed the time course of excitation and inhibition at the focus and the average firing rate in the whole network. We examined three sets of network parameters chosen at random, but leading to different ID thresholds, i.e., no ID generation, high ID threshold (five SimPs) and low ID threshold (three SimPs) (Figures 4B–D respectively). In all cases, excitatory and inhibitory drive increased the firing rate (Figures 4B2,B3,C2,C3,D2,D3). A detailed analysis of the dynamics revealed that after each SimP both excitation and inhibition were strongly but transiently activated (Figures 4B1,C1,D1). An important difference is that, in contrast to inhibition, excitation failed to recover the initial basal conditions, including the simulations in which no ID is generated (Figure 4B). An additional striking difference between the three examples is the maximal inhibition level provided by the inhibitory neurons (the dynamic range). Inhibition reached its highest value in the high ID threshold condition. These results support the view that inhibition strength is a critical factor for focal ID onset. Notably, excitation rose faster than inhibition (slope > 1) driving the growth in firing rate forward. However, the ID occurred only after inhibition reached its maximal value (all inhibitory neurons were active). Therefore the ratio of excitatory versus inhibitory drive and the limiting dynamic range of inhibition are the two critical factors in ID generation. As a summary of results obtained, we report the distribution of points in the excitation-inhibition plane at the focus during the first seven SimPs in 250 Monte-Carlo simulations for the cases that evoked or failed to evoke an ID (normalized by the total area; Figures 4E1,E2). When inhibition at the focus reached high values, no IDs were generated and the ratio between excitation and inhibition remained low. This stands in contrast to the cases which lead to IDs, further supporting the notion that the relationship between excitation and inhibition determines not only the threshold for ID generation, but also whether or not an ID could be evoked. Data obtained from 250 runs also showed a clear correlation between the ID threshold and the average excitation and inhibition in the network during the first SimP (Figures 4F1,F2). This indicates that the overall response of the network, in terms of excitation and inhibition levels, is a good predictor of ID threshold: an increased excitation results in the lowering of the ID threshold and an opposite relationship holds for inhibition.

### Astrocyte-to-neuron signaling decreases the ID threshold

**Figure 5:**
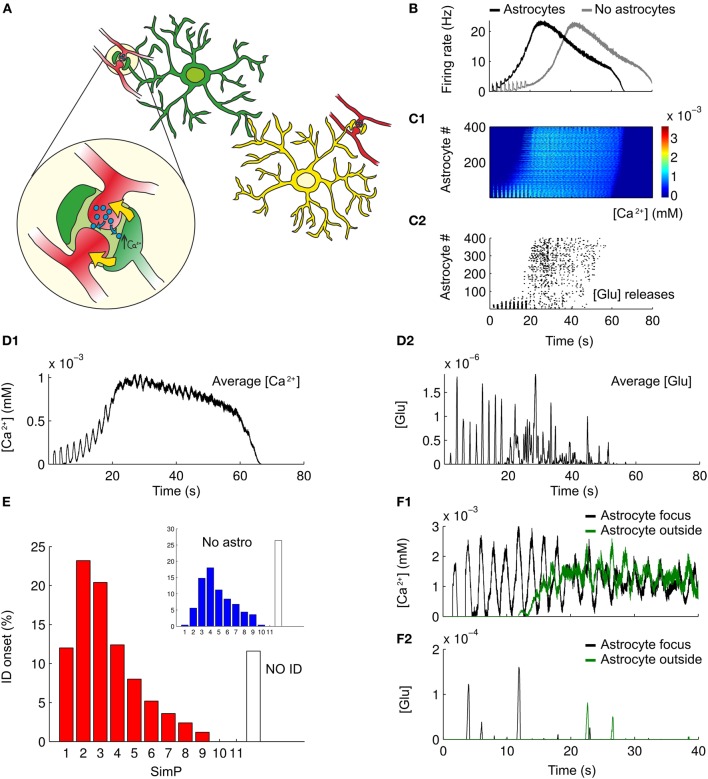
**Astrocytes modulate ID threshold. (A)** A schematic representation of the tripartite synapse and the interplay between astrocytes and neurons in the network. **(B)** Representative average firing rates during simulated IDs with or without astrocytic contribution (black and gray traces respectively). **(C1)** Pseudocolor plot of the Ca^2+^ changes in the astrocytes during a simulated ID. **(C2)** Raster plot of the glutamate released by astrocytes caused by the Ca^2+^ increases as in **C1**. **(D1,D2)** Average trace of Ca^2+^ changes and glutamate released from astrocytes during a simulated ID. **(E)** ID threshold for 250 simulations in a network composed by both neurons and astrocytes. For comparison, panel **D** from Figure 3 has been reproduced as an inset. **(F1,F2)** Representative traces of Ca^2+^ changes and glutamate released from a single astrocytes at the focus (black line) and outside the focus (green line) during a simulated ID.

The model of the single astrocyte (see “Methods”) was incorporated into the network to test how astrocytes may affect ID threshold. Specifically, 400 astrocytes were added to the network model in a parallel 2D sheet of cells (see “Methods”). Astrocytes provide an excitatory feedback to neuronal activity in a Ca^2+^-dependent way (Figure 5A). As illustrated in Figure 5B, in the presence of the astrocyte feedback signal, the ID was evoked by two SimPs, while in its absence a more intense stimulation of neurons was necessary. As illustrated in Figures 5F1,F2 the Ca^2+^ change from a representative astrocyte at the focus was observed to follow rapidly the spiking activity in neurons (Figures 5C1,F1), and astrocytic glutamate release occurred upon the second SimP (Figure 5C2). The average astrocytic Ca^2+^ follow the neuronal activity (example in Figure 5D1) while the average glutamate release occurs transiently (Figure 5D2). The Ca^2+^ change and the release of glutamate from astrocytes outside the focus failed to affect focal ID threshold. The results from 250 Monte-Carlo runs show that the ID threshold was lowered after including the astrocytic feedback signal to neurons (Figure 5E). Once the ID was fully evolved, both the Ca^2+^ change and the release of glutamate from astrocytes within and outside of the focus did not differ significantly (Figures 5F1,F2). However, the initially dominant activity of astrocytes at the focus was followed by an activity of the astrocytes outside the focus that became dominant immediately after the ID onset. These results are consistent with those from slice experiments which showed that when Ca^2+^ elevation in astrocytes from the focus were selectively blocked (by the Ca^2+^ chelator BAPTA) or stimulated (by TFLLR, a peptide agonist of thrombin PAR-1 receptors), the threshold of ID generation increased or decreased, respectively (Gomez-Gonzalo et al., 2010).

### Does an astrocyte inhibitory feedback signal to neurons affect ID threshold?

**Figure 6:**
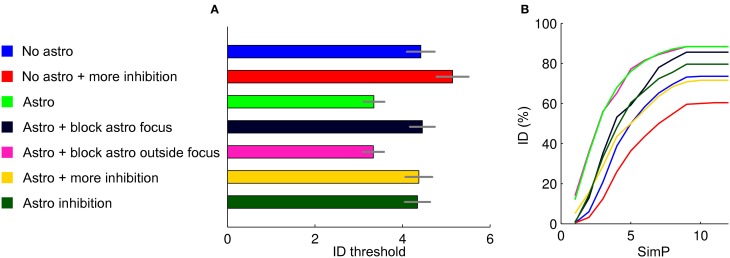
**Summary of the results. (A)** ID threshold histogram in the different conditions. The distributions were described by fitting with Poisson distributions and determining the average values and relative errors. **(B)** Cumulative sum of the ID threshold histograms to show more in details how, for the different conditions tested, the threshold and the number of failures in generating an ID change.

The ID threshold is mainly affected by the interplay between excitation and inhibition. Indeed, as we reported above, ID threshold can be increased by increasing the overall value of the inhibitory activity. The bar graph in Figure 6A reports the results from 250 simulations in different simulation settings, with and without an astrocytic contribution (mean and errors represent the results of a Poisson fit to the ID threshold distributions), while Figure 6B is the cumulative sum of the ID threshold distributions corresponding to the analyzed cases. Blue and red bars show that the ID threshold can be increased by increasing the overall strength of inhibitory connections (in this case from 0.01 to 0.015) in a purely neuronal network (no astrocytes). With higher inhibition, the simulated stimulation failed to induce an ID in 40% of simulations (Figure 6B). As already shown, the introduction of an astrocytic excitatory feedback lowers the ID threshold (green bar) and decreases the number of failures to about 10%. In slice experiments we observed that the inhibition of Ca^2+^ signals in astrocytes at the focus, but not outside the focus, increased the threshold of ID generation. These observations were fully reproduced in the computational model (dark blue and magenta bars) without further manipulations of the model over the results from the previous section. Astrocytic excitatory feedback in a network with stronger inhibitory connections (0.015 as for the red bar) brings back to baseline the ID threshold (yellow).

To explore other factors that may affect ID threshold, we considered the possibility that the activation of astrocytes, or of a subpopulation of astrocytes, results in a release of GABA that can lead to an overall increase of the inhibition strength in the neuronal network. Astrocytes can, indeed, release GABA (Kozlov et al., 2006; Lee et al., 2011; Le Meur et al., 2012). As expected, when we included an astrocytic GABA release in the model, the threshold for ID generation increased (Figure 6, dark green). Note that this was an inhibitory-only feedback involving only GABA release and no glutamate. Surprisingly, however, the threshold for ID generation did not rise over the baseline condition with no astrocytic feedback (blue). A possible explanation for this could be the synchronizing action of an inhibitory GABA signal. Alternatively, the inhibitory feedback signal from astrocytes could be more effective in suppressing inhibitory than excitatory neurons. This action may ultimately generate a new level of complexity in the mechanism that governs the inhibition/excitation balance in the neuron-astrocyte network.

## Discussion

Increasing experimental evidence highlights the physiological significance of the tripartite synapse in which the astrocyte senses neurotransmitter release and, in turn, releases through a Ca^2+^-dependent mechanism gliotransmitters that have feedback modulatory actions on neurons. A number of recent studies *in vitro* and *in vivo* showed that the release of glutamate from astrocytes can, indeed, control both the basal excitability of neurons and some forms of long-term potentiation of synaptic strength (Serrano et al., 2006; Jourdain et al., 2007; Navarrete and Araque, 2010; Santello et al., 2011; Min and Nevian, 2012) and long-term depression (Zhang et al., 2003; Serrano et al., 2006; Han et al., 2012; Min and Nevian, 2012). The contribution of gliotransmission to brain dysfunctions remains, however, poorly understood. A model composed of a network of interacting neurons and astrocytes represents a useful tool in which the spatial-temporal features of focal seizure generation observed in slice models can be replicated and new mechanistic hypotheses can be tested.

Over the last 10 years, different models have been advanced to describe the Ca^2+^ dynamics of astrocytes in response to neuronal signals (Nadkarni and Jung, 2003, 2007; Silchenko and Tass, 2008; Di Garbo, 2009). These biophysical approaches described not only the astrocytic Ca^2+^ response (Li and Rinzel, 1994), but also the possible feedback to neurons. More recently, the possible contribution of astrocytes in events related to the plasticity of synaptic transmission were also included in models (Nadkarni et al., 2008; De Pittà et al., 2011; Wade et al., 2011). While biophysical models are very useful to simulate basic units, like the tripartite synapse, they are hardly suitable for large scale simulations. In contrast, simplified models that include only the basic features of neuron-astrocyte interactions (Postnov et al., 2009) appear more appropriate to describe network dynamics and to investigate the role of astrocytes in epilepsy (Amiri et al., 2012). In our model we did not include any distinct biophysical features that characterize the physiological actions of either neurons or astrocytes. We rather describe the activity of a single astrocyte in terms of the specific input-output signals with which astrocyte and neurons interact. This simplified astrocyte model was then embed in a neuronal network model of IDs based on the Izhikevich's single neuron model.

The slow kinetics of the astrocyte Ca^2+^ response to neuronal activity in the model reflect those of the mGluR-mediated Ca^2+^ elevations that were evoked in astrocytes by axonal afferent stimulation in young rat hippocampal slices (Porter and McCarthy, 1995; Pasti et al., 1997; Perea and Araque, 2005). Indeed, the intracellular Ca^2+^ variations in astrocytes depend primarily, although not exclusively, on activation of metabotropic receptors, phospholipase C-dependent inositol(1,4,5)-trisphosphate (IP3) production and, finally, stimulation of Ca^2+^ release from IP3-sensitive internal Ca^2+^ stores (Kawabata et al., 1996). Glutamate release at the synapse triggers a Ca^2+^ response in astrocytes that increases in both amplitude and frequency of oscillations according to increased levels of the neuronal activity (Pasti et al., 1997). These Ca^2+^ changes trigger a SNARE-dependent exocytosis of glutamate that signals back to affect the excitability of neurons (Araque et al., 2000; Pasti et al., 2001; Parpura et al., 2004). Accordingly, in the model we reproduced the most essential features of glutamate release in response to Ca^2+^ elevations in astrocytes. The release of glutamate is pulsatile and depends on the frequency of Ca^2+^ oscillations (Pasti et al., 2001), while its efficacy is controlled by the amplitude of the Ca^2+^ increase (Parpura and Haydon, 2000). In addition, a steady state Ca^2+^ elevation may trigger only a single episode of release (Pasti et al., 2001).

Some approximations were applied to describe two features that characterize astrocyte signaling in our model. Firstly, we restricted our analysis to somatic Ca^2+^ signals. These Ca^2+^ increases can not be intended to fully represent the synapse-to-astrocyte signaling occurring fundamentally at the proximal and the distal processes that are in contact with the synapse. Indeed, somatic Ca^2+^ increases exhibit a lower frequency and slower kinetics with respect to those at the processes (Di Castro et al., 2011; Panatier et al., 2011). While these recent studies also showed that Ca^2+^ elevations at the astrocytic processes can have a distinct functional role, it is noteworthy that the Ca^2+^ elevation at the soma may represent a response that integrates the signals from the processes where astrocytes sense neurotransmitter release. Accordingly, Ca^2+^ signals at the soma may adequately reflect the overall firing activity of surrounding neurons. Amplitude, frequency and general pattern of somatic Ca^2+^ changes are, indeed, observed to vary according to different levels of neuronal activity (Pasti et al., 1997; Porter and McCarthy, 1997). Secondly, while glutamate (Parpura et al., 1994; Pasti et al., 1997; Bezzi et al., 1998), D-serine (Mothet et al., 2005; Henneberger et al., 2010), ATP (Arcuino et al., 2002; Serrano et al., 2006; Bowser and Khakh, 2007), and GABA (Kozlov et al., 2006; Lee et al., 2011) [for a review see Haydon and Carmignoto (2006)] are the main gliotransmitters mediating astrocyte-to-neuron signaling, in our model we fundamentally focused on glutamate because a large body of information is available about its modulatory action on both basal synaptic transmission (Fellin et al., 2004; Di Castro et al., 2011; Panatier et al., 2011) and long-term plasticity (Zhang et al., 2003; Panatier et al., 2006; Serrano et al., 2006; Jourdain et al., 2007; Navarrete and Araque, 2010; Santello et al., 2011; Han et al., 2012; Min and Nevian, 2012). In addition, the contribution of astrocytic glutamate in some forms of long-term potentiation of synaptic transmission has been recently confirmed in *in vivo* experiments (Takata et al., 2011; Navarrete et al., 2012). The potential role in focal seizure generation of ATP, D-serine and GABA will be the subject of future investigations. It is worth mentioning here that D-serine, and not glycine, is most likely the physiological co-agonist of the synaptic NMDA receptor in the brain (Mothet et al., 2000; Panatier et al., 2006; Fossat et al., 2012; Papouin et al., 2012). Given that D-serine is mainly, although not exclusively (Ding et al., 2011), synthesized in astrocytes and released through a Ca^2+^-dependent mechanism (Wolosker et al., 1999; Wolosker, 2011), astrocytic D-serine may cooperate with glutamate to enhance NMDA receptor openings and through this action favor neuronal excitability ultimately promoting epileptic discharges.

The pathological increase in brain network excitability that eventually leads to focal seizure generation is believed to derive from the activity of excitatory and inhibitory neurons as well as of astrocytes. The cellular events that favor or oppose seizure initiation and propagation remain, however, poorly defined. Our model offers the opportunity to study ID generation in simulated networks composed by either only neurons or interactive astrocytes and neurons. The results that we obtained are summarized in Figure 6 and can be, in our opinion, useful to understand how distinct signaling pathways may govern focal ID generation. Figure 6 plots the average threshold for ID generation in the different conditions (mean ± SD, Figure 6A) and the cumulative sum of the threshold histograms (Figure 6B) showing failures. We found that in a network composed exclusively of neurons an ID can be generated by applying an intense stimulation of a group of neurons. The introduction of astrocytes into the network lowered ID threshold, while the inhibition of astrocyte signaling to neurons within, but not outside the focus, increased ID threshold. These results are fully consistent with those obtained in slice experiments (Gomez-Gonzalo et al., 2010) and demonstrate that focal IDs can be faithfully reproduced in our computational model. Accordingly, our model can be used to make predictions on the distinct contribution of different signaling pathways to ID generation. We present here some results regarding inhibitory signaling pathways. The ID threshold was observed to increase upon procedures that increase the strength of inhibition onto the principal neurons. This was achieved by either increasing the strength of the inhibitory transmission or by including in the model a distinct inhibitory feedback signal from astrocytes to neurons via GABA_A_ receptors. These observations will be useful when addressing in future slice experiments the role of inhibitory signaling in ID generation.

### Conflict of interest statement

The authors declare that the research was conducted in the absence of any commercial or financial relationships that could be construed as a potential conflict of interest.
